# Occult lymph node metastasis is not a favorable factor for resected NSCLC patients

**DOI:** 10.1186/s12885-023-11189-3

**Published:** 2023-09-04

**Authors:** Jing-Sheng Cai, Fan Yang, Xun Wang

**Affiliations:** 1 Department of Thoracic Surgery, Peking University People’s Hospital, No. 11 Xizhimen South Street, Xicheng District, Beijing, 100044 P.R. China; 2 Thoracic Oncology Institute, Peking University People’s Hospital, No. 11 Xizhimen South Street, Xicheng District, Beijing, 100044 P.R. China

**Keywords:** Non-small cell lung cancer, Occult lymph node metastasis, Survivals, Predictive factors

## Abstract

**Background:**

This study was to compare the clinical presentations and survivals between the non-small cell lung cancer (NSCLC) patients with occult lymph node metastasis (OLNM) and those with evident lymph node metastasis (ELNM). We also intended to analyze the predictive factors for OLNM.

**Methods:**

Kaplan–Meier method with log-rank test was used to compare survivals between groups. Propensity score matching (PSM) was used to reduce bias. The least absolute shrinkage and selection operator (LASSO)-penalized Cox multivariable analysis was used to identify the prognostic factors. Random forest was used to determine the predictive factors for OLNM.

**Results:**

A total of 2,067 eligible cases (N0: 1,497 cases; occult N1: 165 cases; evident N1: 54 cases; occult N2: 243 cases; evident N2: 108 cases) were included. The rate of OLNM was 21.4%. Patients with OLNM were tend to be female, non-smoker, adenocarcinoma and had smaller-sized tumors when compared with the patients with ELNM. Survival curves showed that the survivals of the patients with OLNM were similar to those of the patients with ELNM both before and after PSM. Multivariable Cox analysis suggested that positive lymph nodes (PLN) was the only prognostic factor for the patients with OLNM. Random forest showed that clinical tumor size was an important predictive factor for OLNM.

**Conclusions:**

OLNM was not rare. OLNM was not a favorable sign for resected NSCLC patients with lymph node metastasis. PLN determined the survivals of the patients with OLNM. Clinical tumor size was a strong predictive factor for OLNM.

**Supplementary Information:**

The online version contains supplementary material available at 10.1186/s12885-023-11189-3.

## Background

Non-small cell lung cancer (NSCLC) is a leading contributor to cancer-related mortality worldwide [1–3]. Over half of NSCLC patients are diagnosed with advanced diseases, and the 5-year survival rates are dismal. In recent year, occult lymph node metastasis (OLNM), defined as that lymph node metastasis is not detected under clinical evaluations but unexpectedly identified in pathology, has been an active field of research [4–15].

To date, it is evident that the survivals of the patients with OLNM are inferior to those of the patients without lymph node metastasis [7, 13, 15]. However, controversies exist regrading on the prognostic value of OLNM in NSCLC patients with lymph node metastasis. Several clinical series reported a survival benefit associated with OLNM when compared with the evident lymph node metastasis (ELNM) [7, 16]. Yet, there are conflicting data drawing a negative conclusion, revealing that OLNM has little impact on the survivals of the patients [14, 17]. In addition, the number of studies evaluating the clinical presentations and predictive factors for OLNM are modest.

Against this background, this study focused on the resected NSCLC patients with OLNM from a large Chinese cohort. The main object was to systematically characterize and evaluate the survival outcomes of this category of patients. The second object was to explore the predictive factor for OLNM. We anticipated that our study might provide a more comprehensive understanding of this population.

## Methods

### Study design and patient enrollment

Between 1999 and 2018, a series of 7,931 consecutive resected patients were evaluated from our center. The well-managed dataset used in this study was reported before [18]. The included criteria mandated that: (1) confirmed as NSCLC; (2) received surgery and systemic lymphadenectomy. Patients were excluded when meeting the following criteria: (1) sublobar resection; (2) N3 category; (3) M1 category; (4) positive surgical margin; (5) adenocarcinoma in situ; (6) received neoadjuvant therapy; (7) previous or concurrent other cancers; (8) age < 18 years old; (9) unavailable clinicopathological or survival information.

The eligible patients were categorized into five groups: N0 group, occult N1 group, evident N1 group, occult N2 group and evident N2 group according to the results of clinical and pathological results of nodal status.

### Ethic

This study was approved by The Ethics Committee of Peking University People’s Hospital (the approved number: 2020 PHB 421–02). The Ethics Committee of Peking University People’s Hospital waived off the informed consent due to the retrospective nature.

### Nodal status evaluations

In routine, chest and abdomen computed tomography (CT) and brain magnetic resonance imaging (MRI) were performed to determine the clinical tumor-node-metastasis (TNM) stage. Positron emission tomography (PET) imaging was not mandatory in our center because it is expensive and has not been covered by medical insurance in mainland China. In general, lymph node with short axis diameter >  = 1 cm in the CT scan or with maximal standardized uptake value >  = 2.5 in the PET was consider malignant. Once the patients were suspicious of clinical N2 category, invasive nodal evaluation modalities such as endobronchial ultrasonography transbronchial needle aspiration or mediastinoscopy were recommended but not mandatory. After surgery, the formalin-fixed paraffin-embedded tissue sections of harvested lymph nodes with hematoxylin and eosin staining were reviewed by one junior and one senior pathologist of the Department of Pathology in the Peking University People’s Hospital.

### Treatments

The surgical approach and surgical extent were discussed and decided at a multidisciplinary team meeting. All included patients underwent lobectomy or pneumonectomy and systemic lymph nodes dissection. Systemic lymph nodes dissection was defined as mediastinal lymph node dissection of at least three stations, and station 7 (the subcarinal lymph node) must be dissected. Regarding N1 station lymph nodes, the station 10, 11 and 12 were dissected intraoperatively, and the station 13 and 14 were dissected by pathologists from the excised specimen, but this procedure was not mandatory. In addition, at least 6 lymph nodes were harvested. Adjuvant therapies were performed according to the National Comprehensive Cancer Network (NCCN) guidelines [19], usually four cycles of platinum-based doublet chemotherapies were administered to the stage IIA-IIIB NSCLC patients in this period.

### Follow-up

Follow-up information was mainly obtained through telephone calls and hospital visits. In general, postoperative follow-up was performed every three months for the first two years, every six months for the next three to five years and annually thereafter [18]. Physical examinations, tumor markers and chest CT scan were regularly performed at scheduled intervals during follow-up visits. When clinically indicated, brain MRI and bone scans or PET scan were performed.

### Data collection

The clinicopathologic information and treatment data were extracted from the electronic medical record system, which included age, sex, smoking status, family tumor history, body mass index (BMI), comorbidity, staging methods, tumor location, clinical tumor size, clinical tumor (T) category, clinical nodal (N) category, forced expiratory volume in 1 second (FEV1%), diffusion capacity for carbon monoxide (DLCO%), the American Society of Anesthesiologists (ASA) physical status grade, surgical approach, surgical extent, histology, visceral pleural invasion (VPI), lymphovascular invasion (LVI), examined lymph nodes (ELN), positive lymph nodes (PLN), pathologic tumor size, pathologic T category, pathologic N category, pathologic tumor-node-metastasis (TNM) stage, postoperative complications, adjuvant therapy and hospital stay. The 8^th^ edition of the TNM staging manual was used in this study [20]. Complete data analysis was carried out in this study. The endpoints of this study were overall survival (OS) and disease-free survival (DFS). OS was calculated from the date of surgery to the date of death or the last known contact. DFS was calculated from the date of surgery to the date of first recurrence or death. The follow-up information was updated in October 2021.

### Statistically analysis

All statistical analyses were conducted via the R version 4.1.1 (The R Foundation for Statistical Computing, Vienna, Austria; http://www.r-project.org) and the IBM SPSS Statistics (version 25.0, IBM Corp, Armonk, NY, USA). The Shapiro–Wilk test was used to analyze the normal distribution of the continuous variables, and non-normally distributed continuous variables were presented as median (range). The Mann–Whitney U test was used to compare the non-normally distributed variables. Categorical variables were presented as percentages and were compared using Pearson Chi-square test or Fisher’s exact test. OS and DFS were analyzed by the Kaplan–Meier method with log–rank test. A one to one propensity score matching (PSM) was used to reduce the bias caused by the baseline confounders in the occult N1 & evident N1 pair and the occult N2 & evident N2 pair using the R package “MatchIt” [21]. Propensity scores were calculated based on age, sex, comorbidity, surgical approach, surgical extent, histology, pathologic T category, LVI, VPI, complications and adjuvant therapy. The nearest-neighbor matching method with a caliper distance of 0.1 was used in the PSM. A least absolute shrinkage and selection operator (LASSO) method was used to select and minimize prognostic variables using the R package “glmnet” [22]. Variables entered into the LASSO model included age, sex, smoking, family tumor history, comorbidity, BMI, FEV1%, DLCO%, ASA grade, surgical approach, surgical extent, tumor location, histology, VPI, LVI, ELN, PLN, pathologic T category, pathologic N category, complications and adjuvant therapy. The LASSO-selected variables were further entered into a forward stepwise multivariable Cox analysis to determine the final results. Random forest was used to determine the predictive factors for OLNM using the R package “randomForest”. The Variables entered into the random forest included age, sex, smoking, family tumor history, comorbidity, BMI, FEV1%, DLCO%, ASA grade, surgical approach, surgical extent, clinical tumor size, evaluation of nodal status and tumor location. Two-sided *P* < 0.05 was considered statistically significant.

## Results

### Baseline characteristics

A total of 2,067 eligible cases were included in this study. The clinicopathological information are presented in Table 1. The median age was 61 years (range from 22 to 86 years), and over half of cases were male (58.6%). There were 1,497 cases, 165 cases, 54 cases, 243 cases and 108 cases in the N0, occult N1, evident N1, occult N2 and evident N2 group, respectively. Considering the patients with N1 category, there were more females (34.5% vs. 16.7%, *P* = 0.013), non-smokers (43.0% vs. 25.9%, *P* = 0.025) and smaller-sized tumors (*P* = 0.022) in the occult N1 group when compared with those in the evident N1 group. In addition, more patients in the evident N1 group underwent PET (35.2% vs. 19.4%, *P* = 0.009) and open surgery (38.9% vs. 24.8%, *P* = 0.047). Regarding the patients with N2 category, there were more young patients (60 years vs. 63 years, *P* = 0.018), females (35.8% vs. 20.4%, *P* = 0.004) and non-smokers (50.6% vs. 30.6%, *P* < 0.001) in the occult N2 group when compared with those in the evident N2 group. More patients were suffered from preoperative comorbidities in the evident N2 group (65.7% vs. 53.1%, *P* = 0.027). The clinical tumor sizes of the patients with occult N2 metastasis were smaller than those of the patients with evident N2 metastasis (30 mm vs. 35 mm, *P* = 0.002). Adenocarcinoma (ADC) occurred in a sizable fraction of patients with occult N2 metastasis (75.3% vs. 54.6%, *P* < 0.001). Patients in the evident N2 group had more tumors with advanced T categories (*P* = 0.031). After PSM, the variables in the occult N1 & evident N1 pair and the occult N2 & evident N2 pair were all balanced well (Table S1).

**Table 1:** Patient characteristics

Characteristic	N0 (*N* = 1,497)	Occult N1 (*N* = 165)	Evident N1 (*N* = 54)	*P*	Occult N2 (*N* = 243)	Evident N2 (*N* = 108)	*P*
Age, years				0.234^a^			0.018^a^
Median (range)	61 (22–86)	60 (35–81)	63 (45–77)		60 (34–86)	63 (40–81)	
Sex				0.013			0.004
Male	817 (54.6)	108 (65.5)	45 (83.3)		156 (64.2)	86 (79.6)	
Female	680 (45.4)	57 (34.5)	9 (16.7)		87 (35.8)	22 (20.4)	
Smoking				0.025			< 0.001
Non-smoker	934 (62.4)	71 (43.0)	14 (25.9)		123 (50.6)	33 (30.6)	
Smoker	563 (37.6)	94 (57.0)	40 (74.1)		120 (49.4)	75 (69.4)	
Family tumor history				0.194^b^			0.822
Without	1,330 (88.8)	153 (92.7)	53 (98.1)		228 (93.8)	102 (94.4)	
With	167 (11.2)	12 (7.3)	1 (1.9)		15 (6.2)	6 (5.6)	
BMI				0.967^a^			0.372^a^
Median (range)	24.1 (14.3–44.7)	24.4 (16.9–34.6)	24.0 (17.5–33.6)		23.6 (15.4–33.6)	24.2 (17.9–33.3)	
Comorbidity				0.322			0.027
Without	655 (43.8)	80 (48.5)	22 (40.7)		114 (46.9)	37 (34.3)	
with	842 (56.2)	85 (51.5)	32 (59.3)		129 (53.1)	71 (65.7)	
Tumor location				0.952^b^			0.122
RUL	565 (37.7)	57 (34.5)	20 (37.0)		67 (27.6)	39 (36.1)	
RML	113 (7.5)	4 (2.4)	2 (3.7)		13 (5.3)	1 (0.9)	
RLL	282 (18.8)	41 (24.8)	13 (24.1)		56 (23.0)	25 (23.1)	
LUL	316 (21.1)	40 (24.2)	13 (24.1)		63 (25.9)	30 (27.8)	
LLL	221 (14.8)	23 (13.9)	6 (11.1)		44 (18.1)	13 (12.0)	
Clinical T category				0.031^b^			0.050
1a	185 (12.4)	2 (1.2)	1 (1.9)		7 (2.9)	1 (0.9)	
1b	620 (41.4)	43 (26.1)	5 (9.3)		52 (21.4)	16 (14.8)	
1c	364 (24.3)	46 (27.9)	18 (33.3)		78 (32.1)	31 (28.7)	
2a	168 (11.2)	31 (18.8)	10 (18.5)		46 (18.9)	17 (15.7)	
2b	71 (4.7)	25 (15.2)	6 (11.1)		28 (11.5)	14 (13.0)	
3	54 (3.6)	12 (7.3)	10 (18.5)		23 (9.5)	18 (16.7)	
4	35 (2.3)	6 (3.6)	4 (7.4)		9 (3.7)	11 (10.2)	
Clinical tumor size, mm				0.022^a^			0.002^a^
Continue	24 (4–125)	30 (10–130)	34 (8–84)		30 (8–130)	35 (10–104)	
Evaluation of nodal status				0.009^b^			0.122
CT	1,153 (77.0)	132 (80.0)	33 (61.1)		193 (79.4)	80 (74.1)	
PET-CT	334 (22.3)	32 (19.4)	19 (35.2)		47 (19.3)	23 (21.3)	
Invasive modalities	10 (0.7)	1 (0.6)	2 (3.7)		3 (1.2)	5 (4.6)	
FEV1%				0.007^a^			0.608^a^
Median (range)	96.6 (21.1–173.0)	92.8 (46.9–150.0)	84.3 (35.0–125.3)		91.3 (29.4–162.0)	91.4 (51.0–144.4)	
DLCO%				0.091^a^			0.762^a^
Median (range)	87.7 (27.6–147.5)	88.9 (42.7–129.2)	85.1 (11.6–139.1)		84.9 (42.5–160.8)	83.4 (51.1–131.9)	
ASA grade				0.220^b^			0.577
1	296 (19.8)	17 (10.3)	10 (18.5)		50 (20.6)	27 (25.0)	
2	1,146 (76.6)	136 (82.4)	39 (72.2)		188 (77.4)	78 (72.2)	
3	55 (4.5)	12 (7.3)	5 (9.3)		5 (2.1)	3 (2.8)	
Surgical approach				0.047			0.191
VATS	1,376 (91.9)	124 (75.2)	33 (61.1)		189 (77.8)	77 (71.3)	
Open	121 (8.1)	41 (24.8)	21 (38.9)		54 (22.2)	31 (28.7)	
Surgical extent				0.332			0.698
Lobectomy	1,480 (98.9)	151 (91.5)	47 (87.0)		222 (91.4)	100 (92.6)	
Pneumonectomy	17 (1.1)	14 (8.5)	7 (13.0)		21 (8.6)	8 (7.4)	
Histology				0.004			< 0.001
ADC	1,212 (81.0)	89 (53.9)	15 (27.8)		183 (75.3)	59 (54.6)	
SCC	236 (15.8)	66 (40.0)	33 (61.1)		51 (21.0)	39 (36.1)	
Other	49 (3.3)	10 (6.1)	6 (11.1)		9 (3.7)	10 (9.3)	
VPI				0.561			0.885
Negative	1,138 (76.0)	111 (67.3)	34 (63.0)		146 (60.1)	64 (59.3)	
Positive	359 (24.0)	54 (32.7)	20 (37.0)		97 (39.9)	44 (40.7)	
LVI				0.863			0.065
Negative	1,334 (89.1)	103 (62.4)	33 (61.1)		145 (59.7)	53 (49.1)	
Positive	163 (10.9)	62 (37.6)	21 (38.9)		98 (40.3)	55 (50.9)	
ELN				0.664^a^			0.106^a^
Median (range)	16 (6–62)	19 (6–66)	21 (7–47)		17 (6–61)	18 (7–56)	
PLN				0.814^a^			0.239^a^
Median (range)	0 (0–0)	1 (1–8)	1 (1–9)		4 (1–29)	4 (1–26)	
Pathologic tumor size, mm				0.022^a^			0.010^a^
Continue	22 (1–125)	30 (2–105)	30 (8–90)		30 (6–130)	30 (10–100)	
Pathologic T category				0.023^b^			0.031^b^
1a	182 (12.2)	0 (0.0)	1 (1.9)		2 (0.8)	1 (0.9)	
1b	425 (28.4)	25 (15.2)	1 (1.9)		36 (14.8)	6 (5.6)	
1c	244 (16.3)	30 (18.2)	10 (18.5)		37 (15.2)	16 (14.8)	
2a	451 (30.1)	52 (31.5)	19 (35.2)		93 (38.3)	38 (35.2)	
2b	71 (4.7)	27 (16.4)	6 (11.1)		22 (9.1)	14 (13.0)	
3	93 (6.2)	20 (12.1)	10 (18.5)		39 (16.0)	17 (15.7)	
4	31 (2.1)	11 (6.7)	7 (13.0)		14 (5.8)	16 (14.8)	
Postoperative complication				0.161^b^			0.158
Without	1,425 (95.2)	162 (98.2)	51 (94.4)		225 (92.6)	95 (88.0)	
With	72 (4.8)	3 (1.8)	3 (5.6)		18 (7.4)	13 (12.0)	
Adjuvant therapy				0.323			0.560
No	1,189 (79.4)	38 (23.0)	9 (16.7)		41 (16.9)	21 (19.4)	
Yes	308 (20.6)	127 (77.0)	45 (83.3)		202 (83.1)	87 (80.6)	
Hospital stay, day				0.027^a^			0.497
Median (range)	14 (4–58)	14 (6–75)	16 (7–53)		15 (3–38)	15 (2–45)	

*BMI* body mass index, *RUL* right upper lobe, *RML* right middle lobe, *RLL* right low lobe, *LUL* left upper lobe, *LLL* left low lobe, *FEV1* forced expiratory volume in 1 second, *DLCO* diffusion capacity for carbon monoxide, *ASA* American society of Anesthesiologists, *VATS* video-assisted thoracoscopic surgery, *ADC* adenocarcinoma, *SCC* squamous cell carcinoma, *VPI* visceral pleural invasion, *LVI* lymphvascular invasion, *ELN* examined lymph nodes, *PLN* positive lymph nodes, *CT* computed tomography, *PET* positron emission tomography

^a^Mann–Whitney U test

^b^Fisher’s exact test

### Survival analysis

**Fig. 1:**
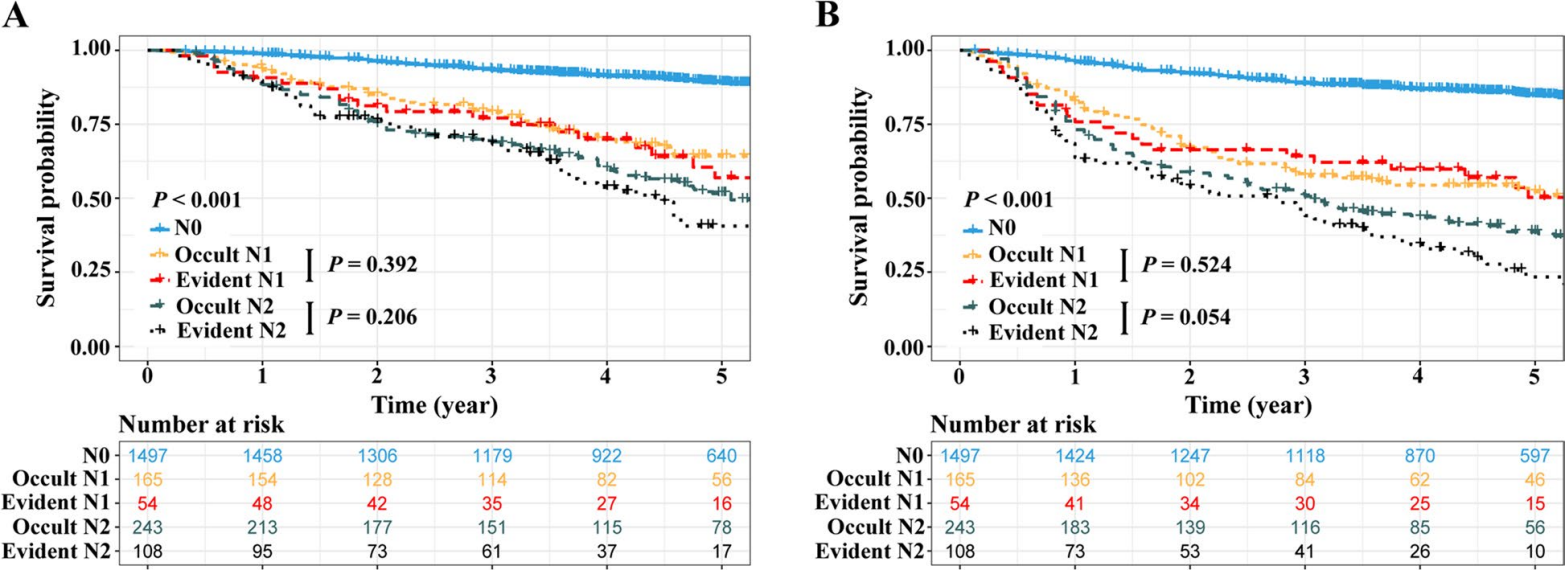
Survivals comparisons among different N categories. **A** OS: N0 vs. occult N1 vs. evident N1 vs. occult N2 vs. evident N2 and (**B**) DFS: N0 vs. occult N1 vs. evident N1 vs. occult N2 vs. evident N2. N, node; OS, overall survival; DFS, disease-free survival; N, node

Kaplan–Meier curves showed that the survivals of the patients with N0 category were superior to that of patients with lymph node metastasis (5-year OS rate: 89.1% vs. 54.0%, *P* < 0.001; 5-year DFS rate: 85.0% vs. 41.1%, *P* < 0.001; Fig. 1). In subgroup analyses, the survivals of the patients with occult N1 metastasis were similar to those of patients with evident N1 metastasis (5-year OS rate: 64.2% vs. 56.9%, *P* = 0.392; 5-year DFS rate: 52.2% vs. 50.3%, *P* = 0.524; Fig. 1). Regarding the patients with N2 category, the OS between the occult and the evident group was comparable (5-year OS rate: 51.6% vs. 40.6%, *P* = 0.206; Fig. 1A). The DFS of the patients with occult N2 metastasis was marginally better than that of the patients with evident N2 metastasis (5-year DFS rate: 38.7% vs. 23.4%, *P* = 0.054; Fig. 1B).

**Fig. 2:**
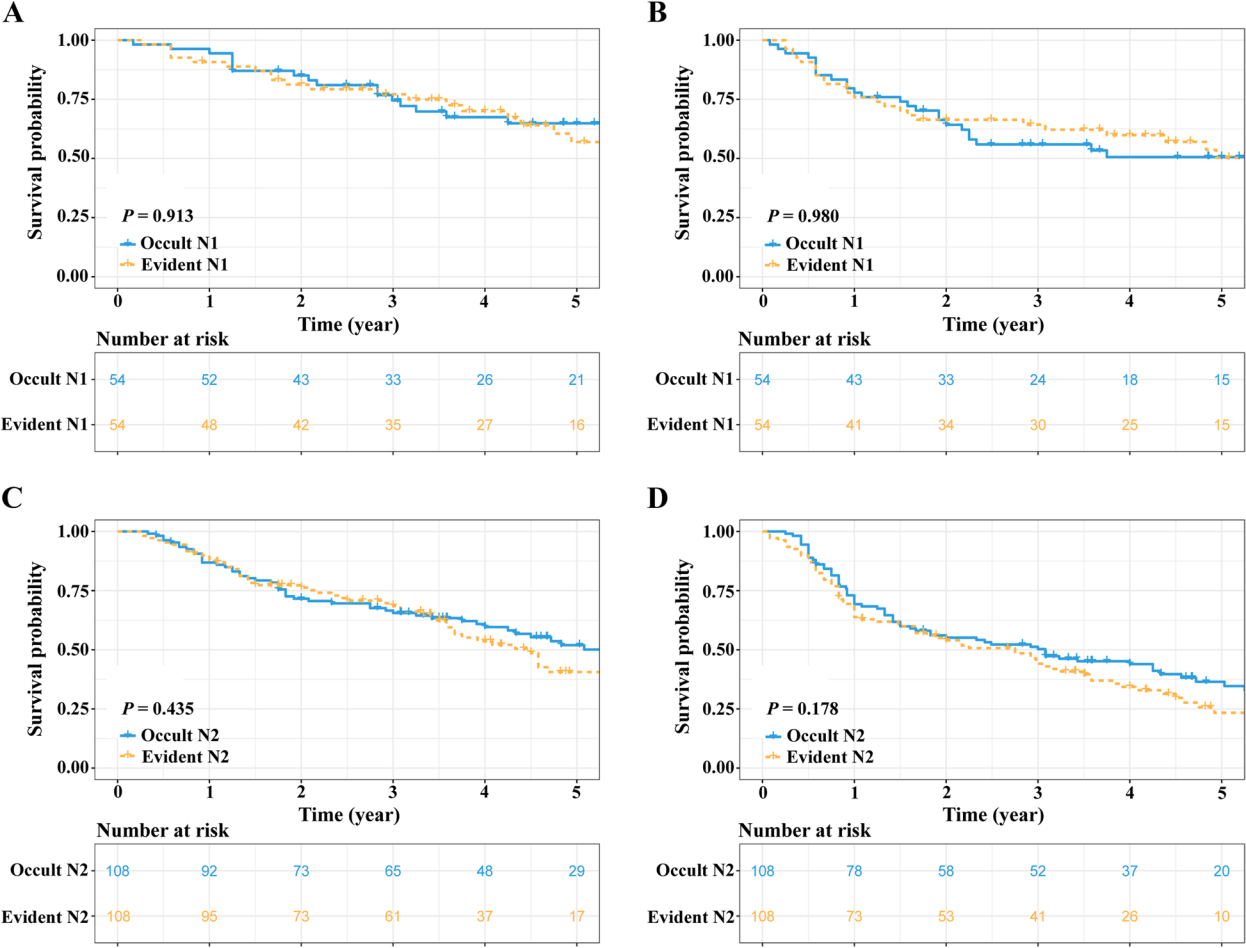
Survivals comparisons between nodal positive patients with and without OLNM after PSM. **A** OS: occult N1 vs. evident N1; (**B**) DFS: occult N1 vs. evident N1; (**C**) OS: occult N2 vs. evident N2 and (**D**) DFS: occult N2 vs. evident N2. PSM, propensity score matching; N, node; OS, overall survival; DFS, disease-free survival; OLNM, occult lymph node metastasis

After PSM, there were 54 and 108 pairs of patients in the occult N1 & evident N1 and the occult N2 & evident N2 group, respectively. Regarding the patients with N1 category, the survival curves showed that these two groups of patients still had similar survival rates (5-year OS rate: 64.8% vs. 56.9%, *P* = 0.913, Fig. 2A; 5-year DFS rate: 50.6% vs. 50.3%, *P* = 0.980, Fig. 2B). Similar results were also observed in the occult N2 & evident N2 matched cohort (5-year OS rate: 52.0% vs. 40.6%, *P* = 0.435, Fig. 2C; 5-year DFS rate: 36.5% vs. 23.4%, *P* = 0.178, Fig. 2D).

### LASSO-penalized multivariable Cox analysis

Considering the entire cohort, LASSO model selected eight potential prognostic factors, including age, smoking, DLCO%, surgical approach, histology, pathologic T category, pathologic N category and PLN, for OS (Figure S1A-B). Accordingly, six potential prognostic factors, including age, smoking, DLCO%, pathologic T category, pathologic N category and PLN, were selected for DFS (Figure S1C-D). Multivariable Cox analysis further confirmed that age (*P* < 0.001), smoking (*P* = 0.001), DLCO% (*P* < 0.001), surgical approach (*P* = 0.012), histology (*P* = 0.034), pathologic T category (*P* < 0.001), pathologic N category (*P* < 0.001) and PLN (*P* < 0.001) were independent prognostic factors for OS (Table S2). Age (*P* < 0.001), smoking (*P* = 0.001), DLCO% (*P* < 0.001), pathologic T category (*P* < 0.001), pathologic N category (*P* < 0.001) and PLN (*P* < 0.001) were proven as the independent prognostic factors for DFS (Table S2).

**Fig. 3:**
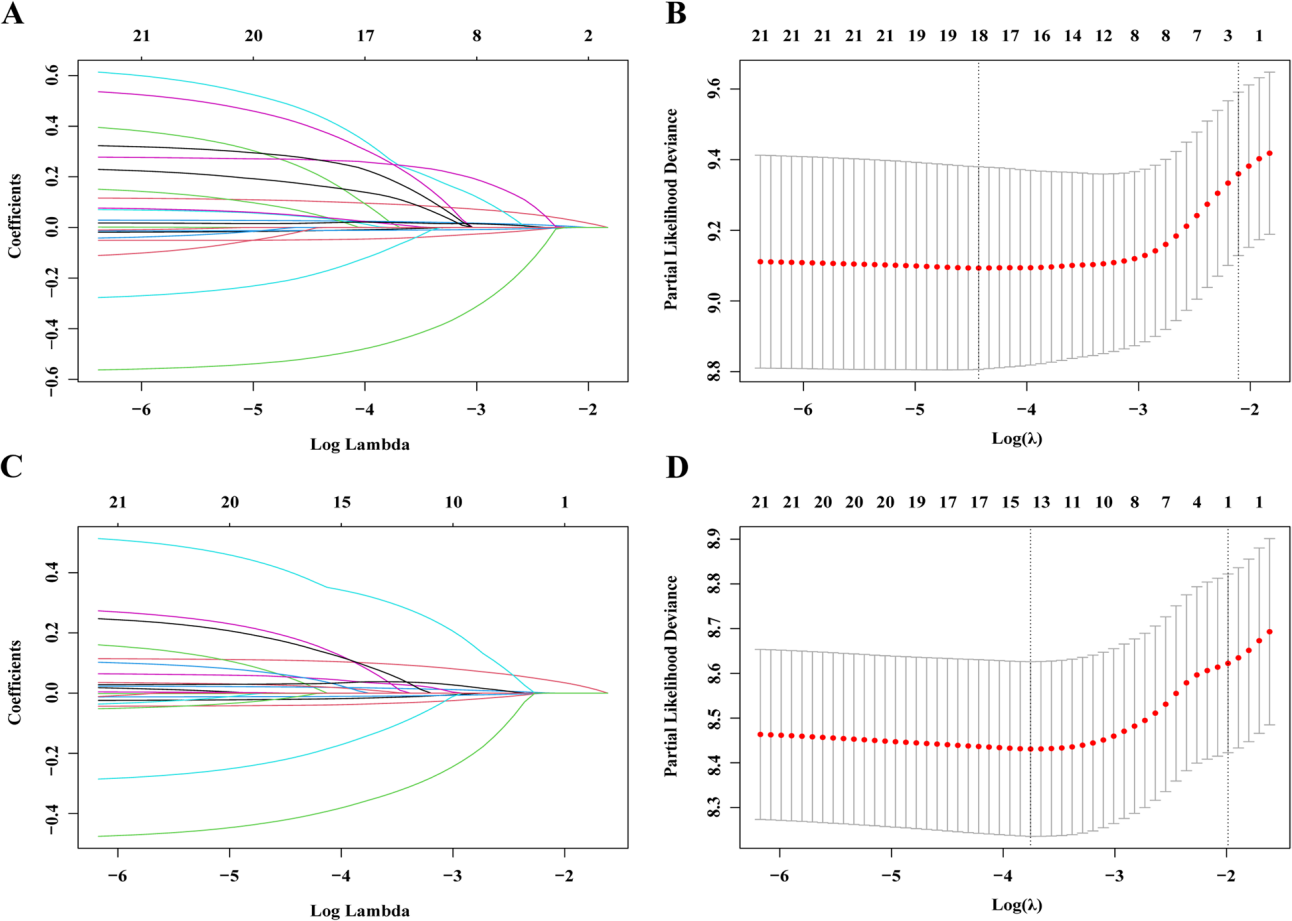
Prognostic factors selection for OS (**A** and **B**) and DFS (**C** and **D**) of the patients with occult lymph node metastasis using the LASSO regression model. LASSO coefficient profiles of 21 included factors against the log (Lambda) sequence for OS (**A**) and DFS (**C**). Tuning parameter (Lambda) selection in the LASSO model used 10-fold cross-validation via minimum criteria (OS: B; DFSS: D). LASSO, least absolute shrinkage and selection operator; OS, overall survival; DFS, disease-free survival

Regarding the patients with OLNM, LASSO model showed that two prognostic factors, including age and PLN, were selected for OS (Fig. 3A, B), and only PLN were selected for DFS (Fig. 3C, D). In further analyses, multivariable Cox analysis confirmed that age (*P* < 0.001) and PLN (*P* < 0.001) were independent prognostic factors for OS, and PLN (*P* < 0.001) was an independent prognostic factor for DFS (Table 2).

**Table 2:** LASSO-penalized multivariable Cox analysis of the patients with occult lymph node metastasis

Characteristic	OS^a^			DFS^b^		
	HR	95% CI	*P*	HR	95% CI	*P*
Age			< 0.001			
Continue	1.091	1.059–1.124				
PLN			< 0.001			< 0.001
Continue	1.033	1.018–1.048		1.089	1.060–1.120	

*OS* overall survival, *DFS* disease-free survival, *PLN* positive lymph nodes

^a^Age and PLN were included in the multivariable Cox analysis of OS

^b^Only PLN was included in the multivariable Cox analysis of DFS

### Random forest

**Fig. 4:**
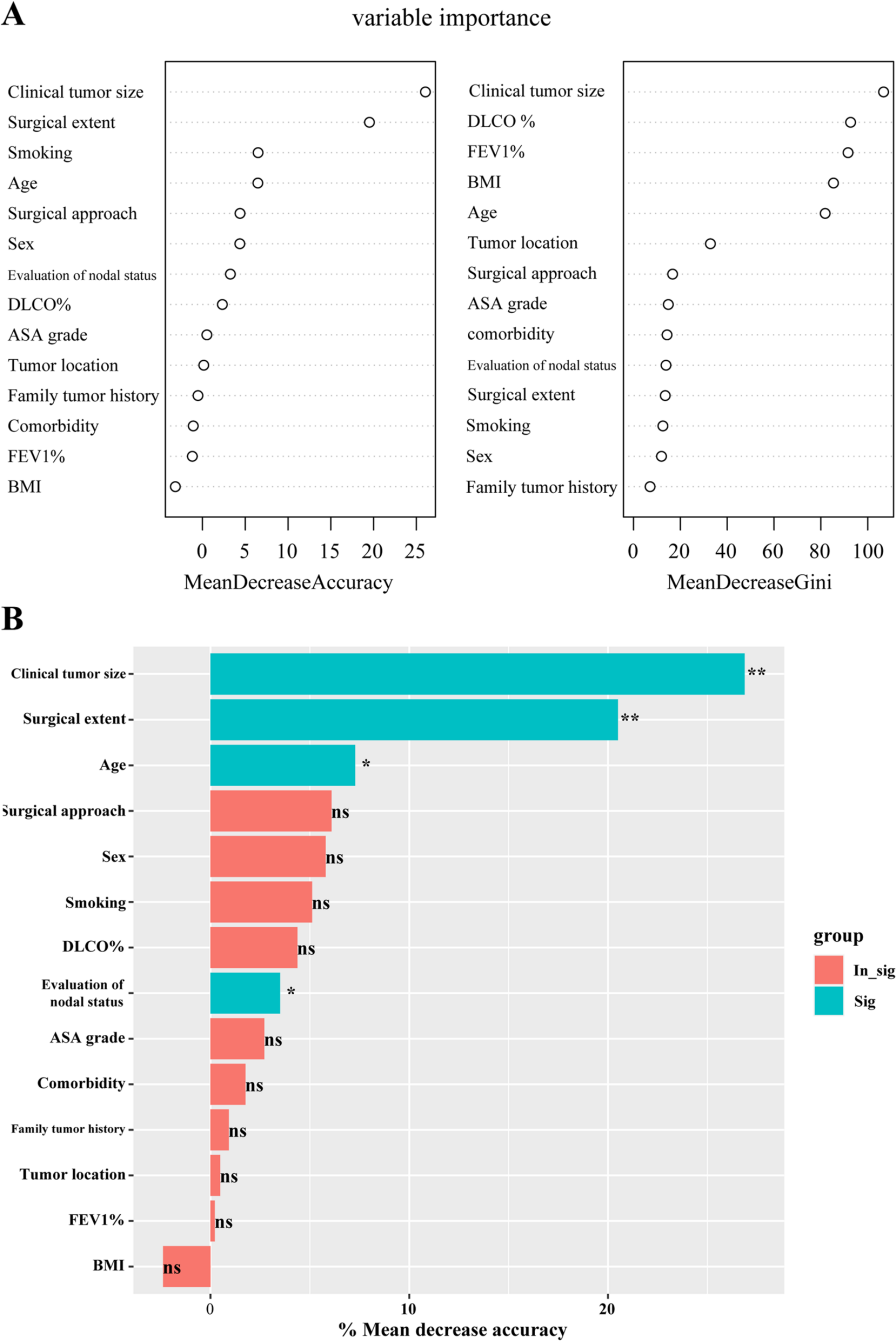
Random forest for selecting the predictive factors of OLNM. **A** the mean decrease accuracy and the mean decrease Gini of each included variables. In general, the greater the indices, the more important the variables and (**B**) the mean decrease accuracy determined the statistically significant variables, ** means *P* < 0.01, * means *P* < 0.05. OLNM, occult lymph node metastasis; FEV1, forced expiratory volume in 1 second; DLCO, diffusion capacity for carbon monoxide; BMI, body mass index; ASA, American society of Anesthesiologists; ns, non-significant

Fourteen pre-incision factors, including age, sex, smoking, family tumor history, comorbidity, BMI, FEV1%, DLCO%, ASA grade, surgical approach, surgical extent, clinical tumor size, evaluation of nodal status and tumor location, were included in the random forest. The results suggested that clinical tumor size (*P* < 0.01) was the strongest predictor, followed by surgical extent (*P* < 0.01), age (*P* < 0.05) and evaluation of nodus status (*P* < 0.05) (Fig. 4).

## Discussion

Our comprehensive analysis of the patient with OLNM demonstrated that the rate of OLNM was not rare (21.4%). Patients with OLNM were tend to be female, non-smoker, ADC and had smaller-sized tumors when compared with the patients with ELNM. Survival curves showed that irrespective of whether it was before or after PSM analyses, the survivals of the patients with OLNM were similar to those of the patients with ELNM. LASSO-penalized multivariable Cox analysis suggested that pathologic N category (N0 vs. occult N1 vs. evident N1 vs. occult N2 vs. evident N2) was a prognostic factor for both OS and DFS in the entire cohort. Only PLN was proven as the prognostic factor for the patients with OLNM. At last, random forest showed that clinical tumor size, surgical extent, age and evaluation of nodal status were the predictive factors for OLNM. Our study provided comprehensive knowledge of NSCLC patients with OLNM and possess a certain clinical reference value.

In forerunning clinical series, Gwozdz et al. [15] and Beyaz et al. [11] reported the frequency of OLNM as 18.9% and 23.1%, which were similar with our results. In other studies however, the authors reported that the rate of OLNM was about 10% [5, 7, 9]. A reason postulated to account for the difference was that in our cohort, a substantial of patients underwent CT scan rather than PET or invasive tools to determine the clinical TNM stage (1591/2067, 77%). It is evidenced that the diagnostic accuracy rate of CT scan is not satisfied [23, 24]. Therefore, high false-negative rate might occur in our cohort. In line with previous studies [7, 8, 25], we demonstrated that patients with OLNM were tend to be female, ADC and had smaller-sized tumors. But the results conflicted with Gwozdz et al.’s study [15], where the authors demonstrated that the incidence of OLNM was not affected by sex, histology and size. In addition, we firstly reported that non-smokers were inclined to have OLNM when compared with the smokers. A possible explanation for this was that there were higher percentage of squamous cell carcinomas (SCCs) in the smoker subgroup (354/425, 83.3%). It is known that SCC is unlikely to metastasize to lymph nodes when compared with ADC.

Previous studies supported the notion that the survivals of the patients with OLNM were superior to those of the patients with ELNM because the former group usually had lower tumor burden [7, 16, 26]. There also have been contrasting reports [14, 17, 24], where the authors argued that whereas clinical underestimation of N category may lead to undertreatment. However, these studies were all suffered from the bias caused by the imbalanced covariates between groups. Our study added to the existing body of evidence on the topic that the survivals of these two groups of patients were comparable. To our best knowledge, this is the first study which compared the survival differences between these two groups using the PSM method. Therefore, our results were more reliable. Herein, we proposed that patients with OLNM should receive the standard treatments and follow-up strategy just like the patients with ELNM.

It is curious to observe that PLN was the only prognostic factor for the patients with OLNM. To date, although tumor burden has not been adopted in the current TNM staging system [20], ample evidences supported that tumor burden is a strong prognostic factor for resected NSCLC patients [27–30]. From our perspectives, PLN was a powerful prognostic factor which might overshadow several conventional prognostic factors such as tumor size, N category and VPI in the patients with positive lymph nodes.

The results of random forest suggested that clinical tumor size was the strongest predictive factor for OLNM, which was confirmed by the previous studies [5, 9]. In the study by Haque et al., the authors directly demonstrated that as the tumor size increases every centimeter, the rate of OLNM increases 10–14% [5]. However, conventional analytic method (logistic regression) was used in their study. As is well known that machine learning algorithms such as random forest specifically suited to figure out associations between data beyond the one-dimensional statistical methods currently used. Thus, our results might be more persuasive. Our finding was important for clinical practice. It is known that several novel treatments such as stereotactic ablative body radiotherapy are potential alternatives to surgical resection for early-stage NSCLC [31]. However, the premise is that the candidate must be nodal negative. Therefore, when encountered large-sized tumors without lymph node involvement, clinicians should be vigilant of the risk of OLNM, and more accurate staging modalities are warranted to exclude lymph node metastasis.

Our study had some limitations. First, only a small portion of patients underwent PET or invasive staging tools (23.1%). Therefore, high false-negative rate of clinical N category might occur in our cohort. In developing countries, preoperative staging by PET or invasive tools is still not common. We hoped patients’ data with definite N category could be applied to validate our conclusion. We optimistically foresaw that with the development of economy and medical technologies, the number of patients with OLNM would de decreased. Second, our study included patients from a time period from 1999 to 2018, which is long time span. There have been huge strides in the development of preoperative evaluation tools and treatment modalities. Therefore, patients in the late period might receive more standard and powerful evaluations and therapies, which could contribute to bias. Third, the variables included in the random forest model were not enough. Further efforts on more detailed information collection such as imaging features and tumor markers are encouraged to improve the performance of the predictive model. Third, although the number of cases included in this study was larger than those of most previous studies, only a small number of patients could be gathered for some subgroups. Therefore, multicenter studies with high quality databases could validate our results. At last, this is a retrospective single-center study, although PSM were performed, the inherent bias was inevitable.

However, notable strengths of this study included that the rigor and efficient statistical methodologies (LASSO model, PSM method and random forest model) used in the study made our results more reliable. The sample size was large, and therefore, our results had certain clinical reference value. In addition, the included patients were treated by a standardized surgical protocol at single institution which could reduce bias. At last, the dataset that we used is well-managed, which includes well-annotated clinicopathologic data and complete follow-up information.

## Conclusions

OLNM was not rare. OLNM was not a favorable sign for NSCLC patients with lymph node metastasis. PLN determined the survivals of the patients with OLNM. Clinical tumor size was a strong predictive factor for OLNM.

### Supplementary Information

**Additional file 1: Table S1.** The covariates distribution in the occult N1 & evident N1 pair and the occult N2 & evident N2 pair after PSM. **Table S2.** LASSO-penalized multivariable Cox analysis of the entire cohort.

**Additional file 2: Figure S1.** Prognostic factors selection for OS (A and B) and DFS (C and D) of the entire cohort using the LASSO regression model. LASSO coefficient profiles of 21 included factors against the log (Lambda) sequence for OS (A) and DFS (C). Tuning parameter (Lambda) selection in the LASSO model used 10-fold cross-validation via minimum criteria (OS: B; DFSS: D). LASSO, least absolute shrinkage and selection operator; OS, overall survival; DFS, disease-free survival.

## Data Availability

Data from this study are available to any interested researchers upon reasonable request to the corresponding author.

## Abbreviations

NSCLC: Non-small cell lung cancer
OLNM: Occult lymph node metastasis
ELNM: Evident lymph node metastasis
CT: Computed tomography
MRI: Magnetic resonance imaging
TNM: Clinical tumor-node-metastasis
PET: Positron emission tomography
NCCN: National Comprehensive Cancer Network
BMI: Body mass index
T: Tumor
N: Node
FEV1: Forced expiratory volume in 1 second
DLCO: Diffusion capacity for carbon monoxide
ASA: American Society of Anesthesiologists
VPI: Visceral pleural invasion
LVI: Lymphovascular invasion
ELN: Examined lymph nodes
PLN: Positive lymph nodes
OS: Overall survival
DFS: Disease-free survival
PSM: Propensity score matching
LASSO: Least absolute shrinkage and selection operator
ADC: Adenocarcinoma
SCC: Squamous cell carcinoma
ns: Non-significant
RUL: Right upper lobe
RML: Right middle lobe
RLL: Right low lobe
LUL: Left upper lobe
LLL: Left low lobe
VATS: Video-assisted thoracoscopic surgery

## References

[1] Siegel RL, Miller KD, Fuchs HE, Jemal A (2021). Cancer Statistics, 2021. CA Cancer J Clin.

[2] Global Burden of Disease Cancer C, Kocarnik JM, Compton K, Dean FE, Fu W, Gaw BL, et al. Cancer Incidence, Mortality, Years of Life Lost, Years Lived With Disability, and Disability-Adjusted Life Years for 29 Cancer Groups From 2010 to 2019: A Systematic Analysis for the Global Burden of Disease Study 2019. JAMA Oncol. 2021;8:420–44. 10.1001/jamaoncol.2021.6987 PMC8719276 34967848

[3] Ganti AK, Klein AB, Cotarla I, Seal B, Chou E (2021). Update of incidence, prevalence, survival, and initial treatment in patients with non-small cell lung cancer in the US. JAMA Oncol.

[4] Mynard N, Nasar A, Rahouma M, Lee B, Harrison S, Chow O (2022). Extent of resection influences survival in early-stage lung cancer with occult nodal disease. Ann Thorac Surg.

[5] Haque W, Singh A, Park HS, Teh BS, Butler EB, Zeng M (2022). Quantifying the rate and predictors of occult lymph node involvement in patients with clinically node-negative non-small cell lung cancer. Acta Oncol.

[6] Dezube AR, Mazzola E, Deeb A, Wiener DC, Marshall MB, Rochefort MW (2022). Mandatory Nodal Evaluation During Resection of Clinical T1a Non-Small Cell Lung Cancers. Ann Thorac Surg.

[7] Deng J, Zhong Y, Wang T, Yang M, Ma M, Song Y (2022). Lung cancer with PET/CT-defined occult nodal metastasis yields favourable prognosis and benefits from adjuvant therapy: a multicentre study. Eur J Nucl Med Mol Imaging.

[8] He XQ, Luo TY, Li X, Huo JW, Gong JW, Li Q (2021). Clinicopathological and computed tomographic features associated with occult lymph node metastasis in patients with peripheral solid non-small cell lung cancer. Eur J Radiol.

[9] Moon Y, Choi SY, Park JK, Lee KY (2020). Risk Factors for Occult Lymph Node Metastasis in Peripheral Non-Small Cell Lung Cancer with Invasive Component Size 3 cm or Less. World J Surg.

[10] Chen Z, Xiong S, Li J, Ou L, Li C, Tao J (2020). DNA methylation markers that correlate with occult lymph node metastases of non-small cell lung cancer and a preliminary prediction model. Transl Lung Cancer Res.

[11] Beyaz F, Verhoeven RLJ, Schuurbiers OCJ, Verhagen A, van der Heijden E (2020). Occult lymph node metastases in clinical N0/N1 NSCLC; A single center in-depth analysis. Lung Cancer.

[12] Ouyang ML, Xia HW, Xu MM, Lin J, Wang LL, Zheng XW (2019). Prediction of occult lymph node metastasis using SUV, volumetric parameters and intratumoral heterogeneity of the primary tumor in T1–2N0M0 lung cancer patients staged by PET/CT. Ann Nucl Med.

[13] Kirmani BH, Volpi S, Aresu G, Peryt A, Win T, Coonar AS (2018). Long term and disease-free survival following surgical resection of occult N2 lung cancer. J Thorac Dis.

[14] Kim MP, Correa AM, Hofstetter WL, Mehran RJ, Rice DC, Roth JA (2018). Occult stage IIIA-N2 patients have excellent overall survival with initial surgery. J Thorac Dis.

[15] Gwozdz P, Pasieka-Lis M, Kolodziej K, Pankowski J, Banas R, Wilkojc M (2018). Prognosis of Patients With Stages I and II Non-Small Cell Lung Cancer With Nodal Micrometastases. Ann Thorac Surg.

[16] Fontaine E, McShane J, Carr M, Shackcloth M, Mediratta N, Page R (2011). Should we operate on microscopic N2 non-small cell lung cancer?. Interact Cardiovasc Thorac Surg.

[17] Yang CF, Kumar A, Gulack BC, Mulvihill MS, Hartwig MG, Wang X (2016). Long-term outcomes after lobectomy for non-small cell lung cancer when unsuspected pN2 disease is found: A National Cancer Data Base analysis. J Thorac Cardiovasc Surg.

[18] Cai JS, Wang X, Yang F, Li Y, Qiu MT (2022). Lymphovascular invasion: A non-sized T descriptor for stage IA non-small cell lung cancer. Thorac Cancer.

[19] National Comprehensive Cancer Network. Non-Small Cell Lung Cancer(Version 4.2021). Availble online at: https://www.nccn.org/professionals/physician__gls/pdf/nscl.pdf. 2021. Accessed 5 Mar 2021.

[20] Goldstraw P, Chansky K, Crowley J, Rami-Porta R, Asamura H, Eberhardt WE (2016). The IASLC Lung Cancer Staging Project: Proposals for Revision of the TNM Stage Groupings in the Forthcoming (Eighth) Edition of the TNM Classification for Lung Cancer. J Thorac Oncol.

[21] Austin PC (2011). An Introduction to Propensity Score Methods for Reducing the Effects of Confounding in Observational Studies. Multivariate Behav Res.

[22] Tibshirani R (1997). The lasso method for variable selection in the Cox model. Stat Med.

[23] Aberle DR, Adams AM, Berg CD, Black WC, Clapp JD, National Lung Screening Trial Research T (2011). Reduced lung-cancer mortality with low-dose computed tomographic screening. N Engl J Med.

[24] Lee DH, Kim JB, Keum DY, Hwang I, Park CK (2013). Long term survival of patients with unsuspected n2 disease in non-small cell lung cancer. Korean J Thorac Cardiovasc Surg.

[25] Gomez-Caro A, Boada M, Cabanas M, Sanchez M, Arguis P, Lomena F (2012). False-negative rate after positron emission tomography/computer tomography scan for mediastinal staging in cI stage non-small-cell lung cancer. Eur J Cardiothorac Surg.

[26] Andre F, Grunenwald D, Pignon JP, Dujon A, Pujol JL, Brichon PY (2000). Survival of patients with resected N2 non-small-cell lung cancer: evidence for a subclassification and implications. J Clin Oncol.

[27] Shang X, Li Z, Lin J, Yu H, Zhao C, Wang H (2020). Incorporating the Number of PLN into the AJCC Stage Could Better Predict the Survival for Patients with NSCLC: A Large Population-Based Study. J Oncol.

[28] Maniwa T, Ohmura A, Hiroshima T, Ike A, Kimura T, Nakamura H (2020). Number of metastatic lymph nodes and zones as prognostic factors in non-small-cell lung cancer. Interact Cardiovasc Thorac Surg.

[29] Katsumata S, Aokage K, Ishii G, Nakasone S, Sakai T, Okada S (2019). Prognostic Impact of the Number of Metastatic Lymph Nodes on the Eighth Edition of the TNM Classification of NSCLC. J Thorac Oncol.

[30] Saji H, Tsuboi M, Shimada Y, Kato Y, Yoshida K, Nomura M (2013). A proposal for combination of total number and anatomical location of involved lymph nodes for nodal classification in non-small cell lung cancer. Chest.

[31] IJ MA, Shoni M, Siegert C, Wiering B, van Engelenburg KCA, Lebenthal A (2019). Survival After Stereotactic Body Radiation Therapy for Clinically Diagnosed or Biopsy-Proven Early-Stage NSCLC: A Systematic Review and Meta-Analysis. J Thorac Oncol.

