# Supplementary material for: Occult lymph node metastasis is not a favorable factor for resected NSCLC patients
Source: BMC Cancer. 2023 Sep 4;23:822. doi: 10.1186/s12885-023-11189-3 (PMC10476354; doi:10.1186/s12885-023-11189-3)
Supplement: Supplementary file 1 — Additional file 1: Table S1. The covariates distribution in the occult N1 & evident N1 pair and the occult N2 & evident N2 pair after PSM. Table S2. LASSO-penalized multivariable Cox analysis of the entire cohort. [file 12885_2023_11189_MOESM1_ESM.docx]

**Table S1. The covariates distribution in the occult N1 & evident N1 pair and the occult N2 & evident N2 pair after PSM.**

| Characteristic | Occult N1  (N=54) | Evident N1  (N=54) | *P* | Occult N2  (N=108) | Evident N2  (N=108) | *P* |
| --- | --- | --- | --- | --- | --- | --- |
| Age, years |  |  | 0.799^a^ |  |  | 0.833 ^a^ |
| Median (range) | 63 (44-79) | 63 (45-77) |  | 64 (38-86) | 63 (40-81) |  |
| Sex |  |  | 0.339 |  |  | 0.376 |
| Male | 41 (75.9) | 45 (83.3) |  | 91 (84.3) | 86 (79.6) |  |
| Female | 13 (24.1) | 9 (16.7) |  | 17 (15.7) | 22 (20.4) |  |
| Comorbidity |  |  | 0.334 |  |  | 0.325 |
| Without | 27 (50.0) | 22 (40.7) |  | 44 (40.7) | 37 (34.3) |  |
| with | 27 (50.0) | 32 (59.3) |  | 64 (59.3) | 71 (65.7) |  |
| Surgical approach |  |  | 0.097 |  |  | 0.880 |
| VATS | 41 (75.9) | 33 (61.1) |  | 78 (72.2) | 77 (71.3) |  |
| Open | 13 (24.1) | 21 (38.9) |  | 30 (27.8) | 31 (28.7) |  |
| Surgical extent |  |  | 0.767 |  |  | 0.251 |
| Lobectomy | 48 (88.9) | 47 (87.0) |  | 95 (88.0) | 100 (92.6) |  |
| Pneumonectomy | 6 (11.1) | 7 (13.0) |  | 13 (12.0) | 8 (7.4) |  |
| Histology |  |  | 0.976 |  |  | 0.816 |
| ADC | 14 (25.9) | 15 (27.8) |  | 63 (58.3) | 59 (54.6) |  |
| SCC | 34 (63.0) | 33 (61.1) |  | 37 (34.3) | 39 (36.1) |  |
| Other | 6 (11.1) | 6 (11.1) |  | 8 (7.4) | 10 (9.3) |  |
| VPI |  |  | 0.841 |  |  | 0.890 |
| Negative | 35 (64.8) | 34 (63.0) |  | 65 (60.2) | 64 (59.3) |  |
| Positive | 19 (35.2) | 20 (37.0) |  | 43 (39.8) | 44 (40.7) |  |
| LVI |  |  | 0.123 |  |  | 0.496 |
| Negative | 25 (46.3) | 33 (61.1) |  | 58 (53.7) | 53 (49.1) |  |
| Positive | 29 (53.7) | 21 (38.9) |  | 50 (46.3) | 55 (50.9) |  |
| Pathologic T category |  |  | 0.525^b^ |  |  | 0.255^b^ |
| 1a | 0 (0.0) | 1 (1.9) |  | 1 (0.9) | 1 (0.9) |  |
| 1b | 4 (7.4) | 1 (1.9) |  | 9 (8.3) | 6 (5.6) |  |
| 1c | 7 (13.0) | 10 (18.5) |  | 13 (12.0) | 16 (14.8) |  |
| 2a | 19 (35.2) | 19 (35.2) |  | 40 (37.0) | 38 (35.2) |  |
| 2b | 10 (18.5) | 6 (11.1) |  | 6 (5.6) | 14 (13.0) |  |
| 3 | 10 (18.5) | 10 (18.5) |  | 28 (25.9) | 17 (15.7) |  |
| 4 | 4 (7.4) | 7 (13.0) |  | 11 (10.2) | 16 (14.8) |  |
| Postoperative complication |  |  | 1.000 |  |  | 1.000 |
| Without | 51 (94.4) | 51 (94.4) |  | 95 (88.0) | 95 (88.0) |  |
| With | 3 (5.6) | 3 (5.6) |  | 13 (12.0) | 13 (12.0) |  |
| Adjuvant therapy |  |  | 0.404 |  |  | 0.615 |
| No | 6 (11.1) | 9 (16.7) |  | 24 (22.2) | 21 (19.4) |  |
| Yes | 48 (88.9) | 45 (83.3) |  | 84 (77.8) | 87 (80.6) |  |

a Mann–Whitney U test

b Fisher’s exact test

VATS, video-assisted thoracoscopic surgery; ADC, adenocarcinoma; SCC, squamous cell carcinoma; VPI, visceral pleural invasion; LVI, lymphvascular invasion; T, tumor

**Table S2. LASSO-penalized multivariable Cox analysis of the entire cohort.**

| Characteristic | OS^a^ | | | DFS^b^ | | |
| --- | --- | --- | --- | --- | --- | --- |
|  | HR | 95% CI | *P* | HR | 95% CI | *P* |
| Age |  |  | <0.001 |  |  | <0.001 |
| Continue | 1.040 | 1.029-1.050 |  | 1.027 | 1.018-1.036 |  |
| Smoking |  |  | 0.001 |  |  | 0.001 |
| Non-smoker | 1 |  |  | 1 |  |  |
| Smoker | 1.424 | 1.154-1.757 |  | 1.342 | 1.129-1.595 |  |
| DLCO% |  |  | <0.001 |  |  | <0.001 |
| Continue | 0.988 | 0.983-0.994 |  | 0.990 | 0.985-0.994 |  |
| Surgical approach |  |  | 0.012 |  |  |  |
| VATS | 1 |  |  |  |  |  |
| Open | 1.344 | 1.067-1.693 |  |  |  |  |
| Histology |  |  | 0.034 |  |  |  |
| ADC | 1 |  |  |  |  |  |
| SCC | 1.003 | 0.785-1.282 |  |  |  |  |
| Other | 1.620 | 1.101-2.385 |  |  |  |  |
| Pathologic T category |  |  | <0.001 |  |  | <0.001 |
| 1a | 1 |  |  | 1 |  |  |
| 1b | 2.820 | 1.017-7.824 |  | 4.569 | 1.665-12.537 |  |
| 1c | 3.324 | 1.195-9.246 |  | 6.356 | 2.319-17.422 |  |
| 2a | 4.403 | 1.618-11.979 |  | 7.506 | 2.774-20.313 |  |
| 2b | 4.862 | 1.732-13.650 |  | 7.946 | 2.860-22.078 |  |
| 3 | 5.016 | 1.794-14.021 |  | 9.279 | 3.368-25.564 |  |
| 4 | 6.576 | 2.300-18.804 |  | 12.059 | 4.295-33.860 |  |
| Pathologic N category |  |  | <0.001 |  |  | <0.001 |
| 0 | 1 |  |  | 1 |  |  |
| Occult N1 | 2.631 | 1.948-3.552 |  | 2.659 | 2.040-3.465 |  |
| Evident N1 | 2.441 | 1.557-3.826 |  | 2.420 | 1.625-3.604 |  |
| Occult N2 | 3.632 | 2.785-4.738 |  | 3.218 | 2.529-4.095 |  |
| Evident N2 | 2.534 | 1.713-3.748 |  | 2.922 | 2.088-4.090 |  |
| PLN |  |  | <0.001 |  |  | <0.001 |
| Continue | 1.066 | 1.040-1.094 |  | 1.077 | 1.052-1.101 |  |

a Age, smoking, DLCO%, surgical approach, histology, pathologic T category, pathologic N category and PLN were included in the multivariable Cox analysis of OS.

b Age, smoking, DLCO%, pathologic T category, pathologic N category and PLN were included in the multivariable Cox analysis of DFS.

OS, overall survival; DFS, disease-free survival; DLCO, diffusion capacity for carbon monoxide; VATS, video-assisted thoracic surgery; ADC, adenocarcinoma; SCC, squamous cell carcinoma; PLN, positive lymph nodes

**Figure legend**

**Figure S1.** Prognostic factors selection for OS (A and B) and DFS (C and D) of the entire cohort using the LASSO regression model. LASSO coefficient profiles of 21 included factors against the log (Lambda) sequence for OS (A) and DFS (C). Tuning parameter (Lambda) selection in the LASSO model used 10-fold cross-validation via minimum criteria (OS: B; DFSS: D). LASSO, least absolute shrinkage and selection operator; OS, overall survival; DFS, disease-free survival
