# Supplementary figures and images for: Occult lymph node metastasis is not a favorable factor for resected NSCLC patients
Source: BMC Cancer. 2023 Sep 4;23:822. doi: 10.1186/s12885-023-11189-3 (PMC10476354; doi:10.1186/s12885-023-11189-3)

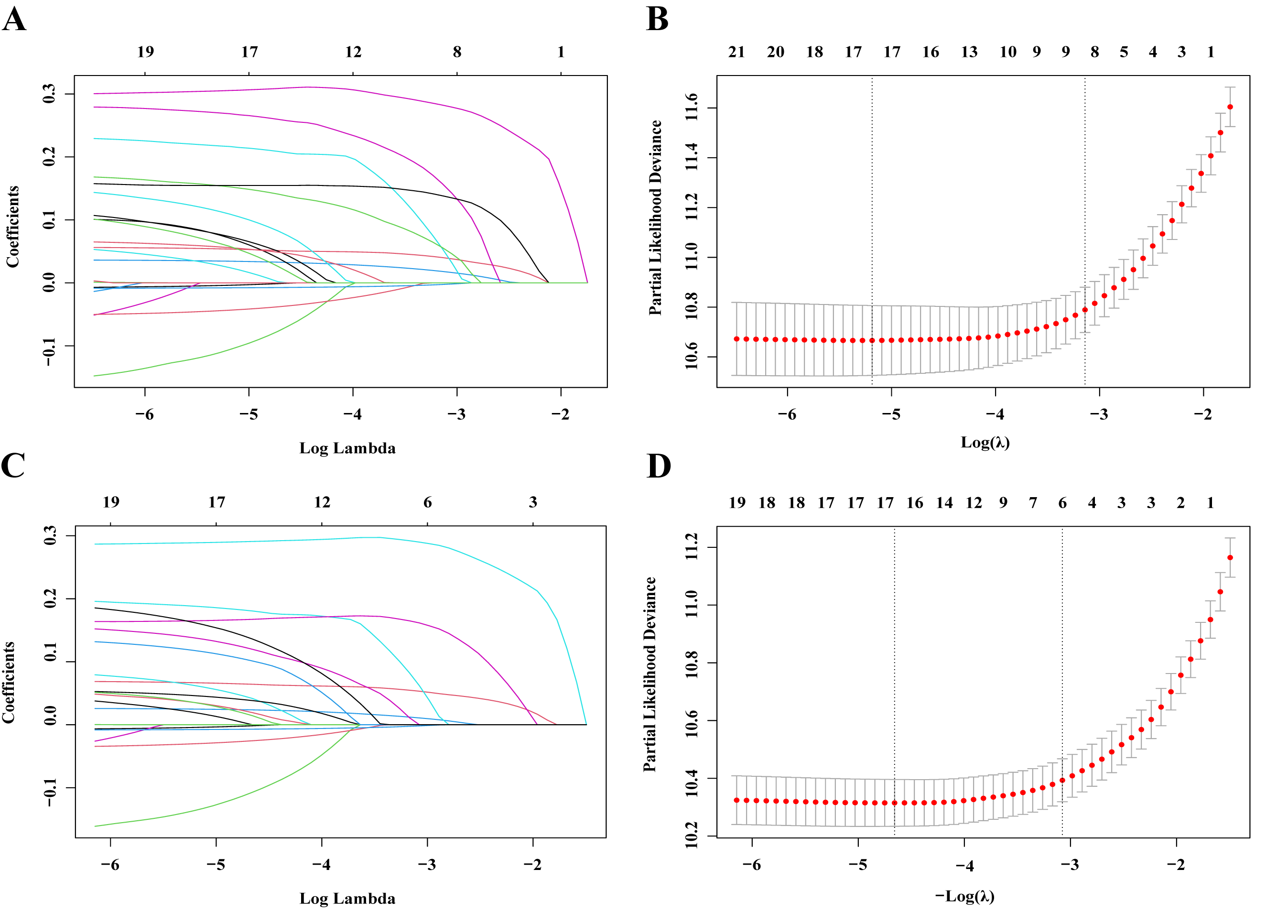

Supplement: Supplementary file 2 — Additional file 2: Figure S1. Prognostic factors selection for OS (A and B) and DFS (C and D) of the entire cohort using the LASSO regression model. LASSO coefficient profiles of 21 included factors against the log (Lambda) sequence for OS (A) and DFS (C). Tuning parameter (Lambda) selection in the LASSO model used 10-fold cross-validation via minimum criteria (OS: B; DFSS: D). LASSO, least absolute shrinkage and selection operator; OS, overall survival; DFS, disease-free survival. [file 12885_2023_11189_MOESM2_ESM.tif]
